# Jaw in a Day: How to Perform Your First Case—Our Workflow

**DOI:** 10.3390/cmtr18030038

**Published:** 2025-09-04

**Authors:** Camilo Mosquera, Hisham Marwan

**Affiliations:** Head and Neck Oncologic Surgery and Microvascular Reconstruction, Department of Surgery, Division of Oral and Maxillofacial Surgery, University of Texas Medical Branch, Galveston, TX 77555, USA; himarwan@utmb.edu

**Keywords:** free tissue flaps, fibula free flap, dental implants, head and neck, reconstructive surgery

## Abstract

Jaw in a Day (JIAD) reconstruction provides immediate restoration of mandibular form and function through a single-stage procedure that integrates fibula free flap reconstruction, virtual surgical planning (VSP), immediate dental implant placement, and delivery of a prefabricated prosthesis. Although the technique provides significant benefits in reducing rehabilitation time and improving patient outcomes, its adoption has been limited due to perceived technical complexity and unfamiliarity with dental workflow. This manuscript provides a detailed, step-by-step protocol to guide surgeons through their first JIAD case, from patient selection and data acquisition to VSP execution, intraoperative coordination, and implant positioning. Emphasis is placed on accurate osteotomy design, implant placement using guided protocols, fabrication of patient-specific hardware, and precise prosthesis pickup techniques. This guide also addresses essential OR team preparation and sterile handling of non-sterile components. By breaking down the process into actionable stages and highlighting common pitfalls and technical tips, this resource aims to lower the barrier for early adopters and enhance the success of initial JIAD cases.

## 1. Introduction

The reconstruction of segmental mandibular defects using fibula free flaps has become a cornerstone of modern head and neck surgery, allowing for reconstructive teams to achieve the goals of mandibular reconstruction by restoring jaw continuity, facial support, and alveolar bone for dental rehabilitation [1]. With the integration of virtual surgical planning (VSP), immediate dental implant placement, design of custom reconstruction plates, and 3D-printed dental prosthesis delivery, the concept of “Jaw-in-a-Day” (JIAD) has emerged as a transformative technique [2,3]. This approach enables the simultaneous restoration of mandibular continuity, function, and esthetics in a single operative stage, reducing overall rehabilitation time and improving quality of life.

Despite its benefits, the JIAD protocol remains underutilized, mainly due to its perceived complexity, cost, and the steep learning curve associated with multi-team coordination, digital planning, and intraoperative execution. For many surgeons unfamiliar with the dental aspect of the reconstruction, their first case can be daunting. Without adequate preparation and insight into the process, first-time attempts can be prone to avoidable errors.

The purpose of this article is to provide a comprehensive step-by-step guide for performing a surgeon’s first fibula JIAD reconstruction. Our specific aim is to outline the essential elements of preoperative planning, virtual surgical design, intraoperative execution, and postoperative care, while highlighting key technical tips, common pitfalls, and logistical considerations to help early adopters avoid complications and achieve successful outcomes.

## 2. Materials and Methods

The authors categorized the steps for the JIAD procedure into three categories: Preoperative planning, Intraoperative execution, and postoperative care.

## 3. Results

### 3.1. Preoperative Planning

Preoperative evaluation is crucial in determining whether a patient is a suitable candidate for reconstruction using a free fibula flap following segmental mandibulectomy. Many patients requiring this procedure are older and have multiple comorbidities, making medical optimization before surgery critically important. Additionally, preoperative planning must include an assessment of the anatomical variations in the lower leg, its arterial vascularization, and any arteriosclerosis, as these factors may contraindicate the use of the free fibula flap (FFF). Such conditions can increase the risk of vascular compromise to the foot and distal lower extremity [4].

CT-Angiography (CTA) of the bilateral lower extremities is the gold standard for vascular assessment. It is essential for virtual surgical planning and creating 3D-printed custom cutting guides and custom reconstruction plates [5]. The success of the JIAD reconstruction relies on the accuracy of the osteotomies in the fibula and the resection site, which can only be achieved by using custom-made devices, patient-specific implants, and stereolithographic models during the surgical procedure.

#### 3.1.1. Data Gathering

To obtain high-quality and fidelity in custom-made implants and guides, it is essential to have high-quality imaging and data. Low-quality data will produce low-quality products, leading to inaccurate and unsuccessful JIAD reconstruction.

The necessary data to be collected (Table 1) is a maxillofacial CT-scan with 1 mm thickness cuts and increments, CTA Bilateral lower extremities with 1 mm cuts and increments, and an STL file of the teeth that should be ideally obtained with an intraoral scanner, or by obtaining dental impressions and using a desktop scanner to digitalize the teeth. These files are then sent to the vendor for use during the virtual surgical planning (VSP) meeting.

#### 3.1.2. VSP Meeting

During the VSP meeting, the dental data are superimposed with the CT maxillofacial data to increase the accuracy of the fabricated guides [6]. This dental data is also used to 3D print the temporary dental prosthesis that will be placed intraoperatively. In the case of complete edentulous patients, where a full arch reconstruction is planned, the vendor can idealize the dentition and virtually position the occlusion.

The VSP starts with the simulated jaw resection. The surgical team determines the bony margins for the surgical resection; the authors recommend resecting enough to fit fibula segments of at least 2.5 cm. In the posterior mandible, it is recommended to include the coronoid process in patients with cancer or osteoradionecrosis (ORN) to prevent or improve trismus. For mandibular body defects, it is recommended to extend the osteotomy up to the first premolar or canine, if the margin allows, designing the osteotomy through the socket rather than between teeth, to maintain bone on the remaining teeth. For defects crossing the midline, consider including all four incisors to allow enough space for a sufficiently large anterior fibula segment.

**Figure 1:**
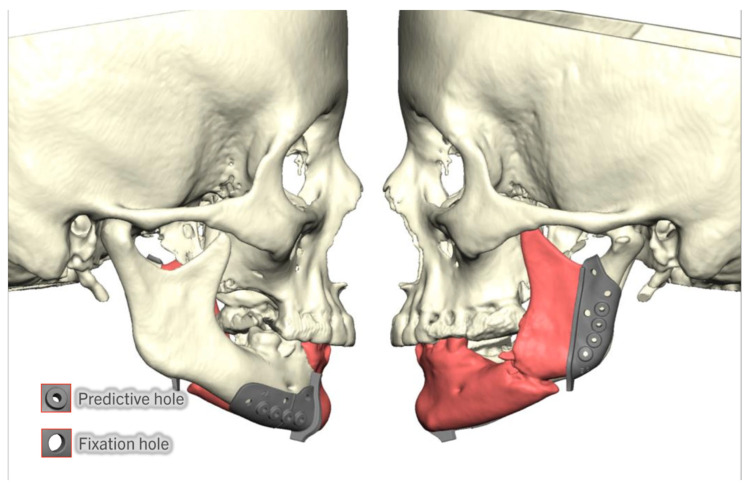
Individual mandibular cutting guides with registration points in the posterior border of the mandibular ramus, sigmoid notch, mental foramen, and inferior border of the mandible. Area highlighted in red corresponds to planned resection.

The mandibular resection cutting guides can be designed as one bridged guide or as distinct, independent guides. The authors typically design individual guides to facilitate smaller incisions in the neck or, on occasion, intraoral resections of the tumors. It is essential to note that a single bridged piece enhances the accuracy of the resection by reducing the likelihood of mispositioning the cutting guide. In contrast, the independent guides have the potential to slide over the bone and be placed in the incorrect position. To prevent this, it is essential to use anatomical landmarks as registration points to set the guides precisely (Figure 1).

**Figure 2:**
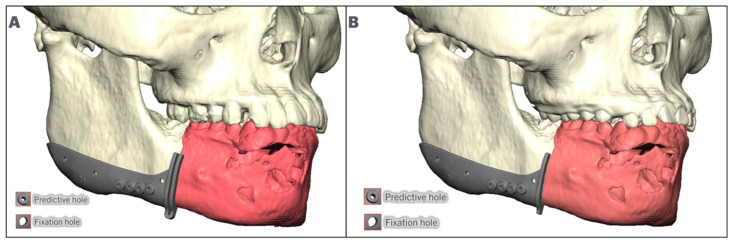
Example of two resection cutting guide designs. (**A**): Cutting guide with slots design. (**B**): Cutting guide with wall design. Area highlighted in red corresponds to planned resection.

The resection cutting guides can also be designed to have slots or cutting walls to fit the saw for the osteotomies (Figure 2). The authors believe that using the wall design increases visibility at the osteotomy site, allowing for the surgeon to make sure that the saw is seated passively against the wall. This allows for the accurate reproduction of the osteotomy angle and avoids interferences during the fibula inset.

The right or left fibula is selected based on vascular supply and the surgeon’s preference. The lateral face of the fibula is used as the plating surface, and the anterior surface of the fibula is used to place the dental implants. However, the authors have used the posterior surface of the fibula when the case does not allow otherwise, particularly in bone-only reconstructions. The vascular pedicle can be oriented anteriorly or posteriorly, depending on the surgeon’s preference and the availability of receptor vessels. If a skin paddle is to be used, it is recommended to design it to come over the buccal surface, thereby recreating a vestibule.

**Figure 3:**
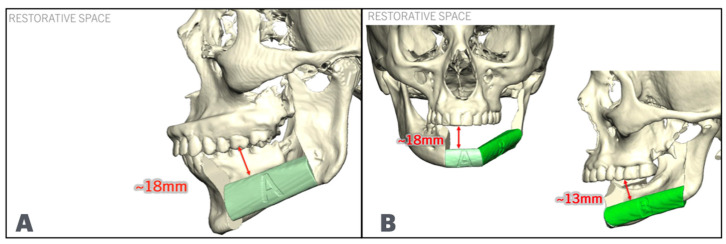
Fibula (In green) positioning for mandibular defects, achieving enough restorative space. (**A**): Fibula segment aligned with the inferior border posteriorly and raised anteriorly. (**B**): Anterior fibula segment aligned with inferior border, and posterior fibula segment lowered to allow for minimal restorative space.

**Figure 4:**
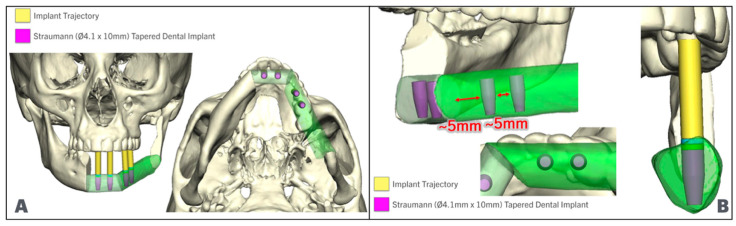
Implant positioning in fibula segments (In green), (**A**): Implant parallelism in anterior and lateral fibula segments. (**B**): Implants are positioned allowing for 5 mm between implant and osteotomy, and 5 mm between implants while maintaining parallelism and implant trajectory aligned with the occlusal surface.

The fibula position is maintained low and aligned with the inferior border of the posterior mandible. It is raised anteriorly at the symphysis/parasymphysis level to allow for 13–18 mm of restorative space. In some cases where the vertical facial height is decreased, it may be necessary to place the fibula aligned with the inferior border anteriorly or displace the posterior segment lower than the mandibular angle to allow for enough restorative space, while maintaining bone-to-bone contact (Figure 3). Once the position of the fibula has been defined, the dental implants are placed. The authors recommend placing at least two implants at each fibula segment, oriented in such a way that they emerge lingual or at the level of the occlusal surfaces of the teeth, avoiding extreme angulation towards the lingual or buccal plate. The parallelism of the implants is checked to ensure a passive insertion path for the temporary prosthesis. The authors recommend maintaining a distance of at least 5 mm from the osteotomy and 5 mm between implants (Figure 4).

The fibula cutting guides are designed with an implant-restrictive positioning guide incorporated. This implant positioning guide enables the placement of dental implants during the harvesting of the flap, and it is restrictive, meaning that it prevents the surgeon from accidentally modifying the angle of drilling or implant insertion. The implant placement area in the fibula cutting guide must have a diameter that matches the sleeves of the guided implant kit that the surgeon prefers. The authors typically use the Straumann Bone-Level Tapered (BLT) Implant System and have found that most cases are suitable for using 4.1 × 10 mm or 4.1 × 12 mm BLT implants.

**Figure 5:**
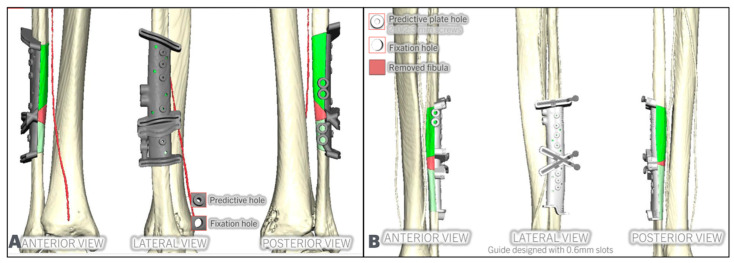
Examples of fibula cutting guides made in titanium. The planned fibula segments are shown in green (**A**), and polyamide with titanium sleeves (**B**). Note how the implant guides are incorporated into both fibula cutting guides.

Both the mandible and the fibula cutting guides can be designed in polyamide or titanium. The authors recommend using titanium guides for both resection and flap; however, if Polyamide guides are used, metal sleeves are highly recommended for the fibula guide to allow for a more precise cut (Figure 5).

**Figure 6:**
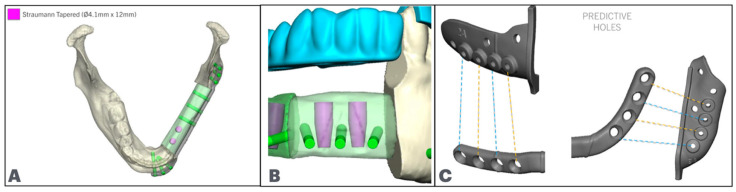
(**A**): Fixation screws posterior to dental implants. The planned fibula segments are shown in green. (**B**): Fixation screws in between implants. (**C**): Cutting guides predictive holes match the fixation screws.

The patient-specific plate is designed next, located low on the fibula segments, with the fixation holes placed in between or behind the implants. In the native mandible, the number of fixation holes follows the reconstructive plating principles. The reconstruction plate holes are designed to match the predictive holes of the resection cutting guide (Figure 6).

**Figure 7:**
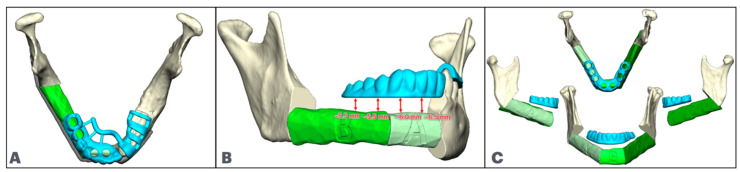
(**A**): Floating temporary prosthesis shown in blue. (**B**): Space between the temporary prosthesis and the fibula segment. (**C**): Floating prosthesis in full arch reconstruction. The planned fibula segments are shown in green.

Once the resection and the fibula guides are designed, the dental data is used to create a 3D reconstruction of the dentition. The temporary prosthesis can be designed as a floating device if enough native teeth are still present. This floating denture serves as a splint, enabling the precise positioning of the denture over the fibula and maintaining an adequate vertical position of the denture in relation to the implants. In the case of a total arch reconstruction, where no native dentition remains, the authors use a maxillary Stealth model and an articulated maxillary model, designed by Khatib et al. and referred to as the “Fibiculator [7].” The stealth model is used to hold the temporary prosthesis in place and correctly position the prosthesis vertically. The maxillary articulated model is used to verify occlusion after the prosthesis is picked up. The temporary prosthesis is designed with enough space between the lower surface and the fibula to allow for closure and hygiene after surgery (Figure 7).

**Figure 8:**
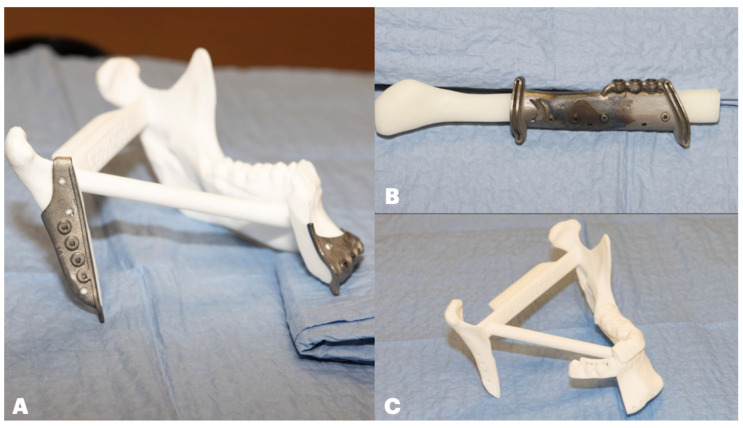
(**A**): Mandibular resected model with cutting guide in position. (**B**): Fibula cutting guide with incorporated dental implant guide. (**C**): Verification model of 3D-printed prosthesis over mandibular resected model.

After the VSP meeting, the surgeon should request the following devices to be available in surgery (Figure 8).

**Figure 9:**
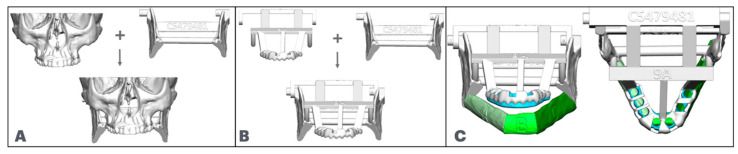
(**A**): Articulated maxillary model. (**B**): Stealth maxillary articulated model. (**C**): Stealth maxillary model holding the temporary prosthesis.

○ Patient-specific reconstruction plate;
○ Patient-specific resection cutting guides;
○ Patient-specific fibula cutting guide with implant guide incorporated;
○ Resected model with fixation holes to secure the reconstruction plate;
○ Temporary dental prosthesis;
○ Stealth maxilla and articulated maxillary model for total arch reconstructions (Figure 9).

### 3.2. Intraoperative Execution

#### 3.2.1. Operating Room (OR) Staff Training

Performing a successful first JIAD reconstruction requires not only surgical expertise but also a highly coordinated and knowledgeable OR team. Because this procedure uniquely integrates microvascular free flap techniques with immediate dental implant placement and prosthetic workflows, it presents novel challenges for OR staff who may be unfamiliar with the specialized dental implant components and materials involved.

Many OR nurses, scrub technicians, and circulating staff have limited prior experience with dental implant systems. This encompasses a wide range of components, including implant fixtures, multi-unit abutments, impression copings, and prosthetic parts. Furthermore, materials such as acrylic resin used for prosthesis fabrication, along with the specific handling and sterilization requirements, often fall outside their regular practice. This lack of familiarity can result in delays, an increased risk of contamination, or unintentional damage to costly components.

The JIAD procedure involves introducing non-sterile components into the surgical field, such as 3D-printed prostheses, acrylic tip applicators, and impression coping abutments. This can raise concerns among OR staff unfamiliar with dental workflows, particularly regarding the risk of cross-contamination [8]. To address this, the authors follow an institutional protocol where these items are submerged in povidone-iodine for 10 min before use. The acrylic tip applicator is enclosed in a sterile plastic bag, allowing intraoperative application while preserving sterility. Clear protocols like this are essential to maintain aseptic technique and reduce anxiety among inexperienced OR teams.

To address these challenges, it is essential to conduct dedicated preoperative training sessions for OR staff. These sessions should include hands-on familiarization with implant kits and their components, detailed instruction on aseptic protocols for handling non-sterile parts, and clear labeling and organization of implant surgical trays. Involving dental implant representatives in these training sessions can boost staff confidence and ensure proper handling of components. Additionally, assigning a dedicated surgical technologist or circulating nurse with specialized training in implant workflows during the first few JIAD cases can enhance efficiency and reduce the likelihood of intraoperative errors.

#### 3.2.2. Surgical Procedure

The authors usually perform the procedure in a two-team approach with the ablative and reconstructive teams working in parallel. Following airway protection, the head and neck team initiates the procedure with neck dissection and tumor ablation, utilizing patient-specific resection cutting guides designed during virtual surgical planning. Care is taken to precisely position the cutting guide using the anatomic landmarks planned as registration points during the virtual surgical meeting. This part of the procedure is essential, as failure to position the guides correctly will result in a larger or smaller defect, where the fibula segments will not fit properly.

The resection cutting guides are fixated, and the predictive holes are drilled. Care is then turned to perform the osteotomies. This is a deceptive, simple step; however, care must be taken to accurately reproduce the same angle with the saw that the cutting guide has. To achieve this, the authors emphasize maintaining the saw blade flush and parallel to the cutting guide wall. Separating the saw blade from the guide wall will result in a different angle that will generate an interference point with the fibula segment.

**Figure 10:**
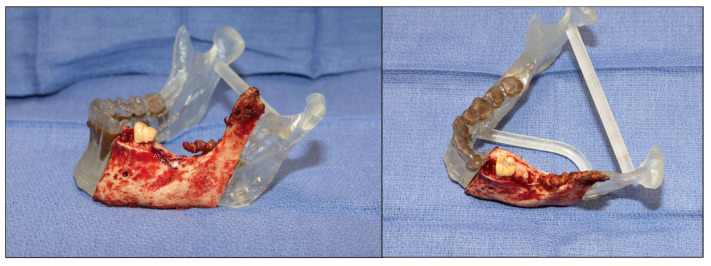
Specimen on the resected model to verify the accuracy of osteotomies.

Once the resection is complete, the authors recommend verifying the accuracy of the resection by using the resected 3D printed model (Figure 10). This allows the ablative team to eliminate any identified interferences before the reconstructive team enters ischemia time.

Simultaneously with the resection, the reconstructive team harvests the FFF in the standard fashion [9]. If a skin paddle is anticipated, it is desirable to position it buccally as it will create a vestibule. A limited skin paddle is advisable in cases with minimal soft tissue defects. The authors typically prefer primary closure and a bone-only flap in cases with limited soft tissue defects. After the proximal and distal fibular osteotomies are completed, the fibula has been mobilized laterally. The pedicle has been traced proximally to the trifurcation; attention is turned to the osteotomies and placement of the dental implants. The fibula cutting guide is positioned and secured with screws. Care is taken to verify that the dental implant guides are located over the anterior surface with enough available bone. Next, the dental implants are placed. The recommended hard-bone protocol from the implant company must be followed, and the implant drilling sequence is completed in the standard fashion with controlled irrigation, speed, and torque. Due to the hardiness of the fibula bone, the authors recommend bi-cortical drilling.

In addition, the authors recommend using countersinks or increasing the drill size by one, only on the outer cortex, to allow for passive implant placement while preserving immediate retention. The authors usually place Bone-Level implants one millimeter sub-crestal, taking care to avoid bi-cortical implant anchorage. Although not yet supported by definitive clinical evidence, some surgeons intentionally avoid bicortical anchorage when placing dental implants into the fibula, based on the belief that this approach may reduce the risk of fibula segment fracture in the event of peri-implantitis extending to the implant apex. It is important to note that this theoretical concern remains anecdotal and warrants further investigation.

**Figure 11:**
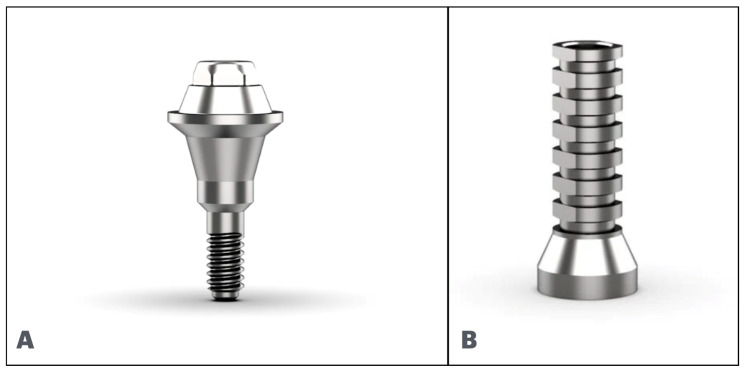
(**A**): Multi-unit abutment. (**B**): Temporary coping abutment.

Once the implants are placed and the fibular closing osteotomies are completed, the cutting guide is removed, allowing access to the dental implants. At this point, straight multi-unit abutments are used to raise the prosthetic platform, and then the straight coping abutments are placed. The authors usually use a 4 mm straight multi-unit abutment and a straight temporary coping abutment (Figure 11).

**Figure 12:**
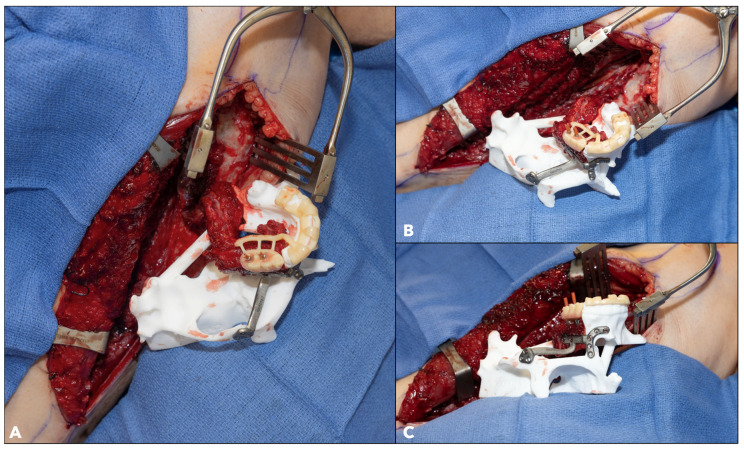
Example of Maxillary Jaw in a Day. (**A**): Emergence of implants’ temporary coping abutments aligned with perforations on the occlusal surface of the dental prosthesis. (**B**): Passive position of the fibula segment on the resected model with good fit and no interferences. (**C**): Verification of passive inset of fibula segment and correct vertical position of dental prosthesis.

Once the multi-unit and temporary coping abutments have been placed, the custom-made reconstruction plate is secured to the resected model using the screws removed from the fibula cutting guide. Next, the fibula flap is adapted to the resected model and loosely fixed to the plate, allowing for slight rotational movement of the segments. At this point, the 3D printed floating temporary prosthesis is placed over the resected model dentition, and the temporary abutments are aligned with the occlusal opening prepared for the emergence of the implants’ abutments. It is common to see at this step that the abutments are hitting against the lingual or buccal surface of the temporary prosthesis; this is usually due to a “barrel-roll” effect on the fibula segment that can be solved by rotating it buccally or lingually as needed until the abutments are located passively on the prosthesis openings. Suppose the temporary prosthesis is not passively aligned with the temporary abutments and is forced into position; the patient will have a malocclusion once the flap is transferred to the head. The authors recommend achieving the best and most passive positioning and insertion pathway of the fibula on the model and the prosthesis on the implants’ abutments. Once the position of the fibula segments and the emergence of the implants’ abutments on the temporary prosthesis have been confirmed, the fibula fixation screws are tightened, taking care to prevent rotation of the bony segment as the screws secure the bone to the plate. Once this is complete, the opening of the temporary coping screw is covered with a small red rubber catheter to prevent acrylic inflow during the prosthesis pickup (Figure 12).

#### 3.2.3. Temporary Prosthesis Pickup

**Figure 13:**
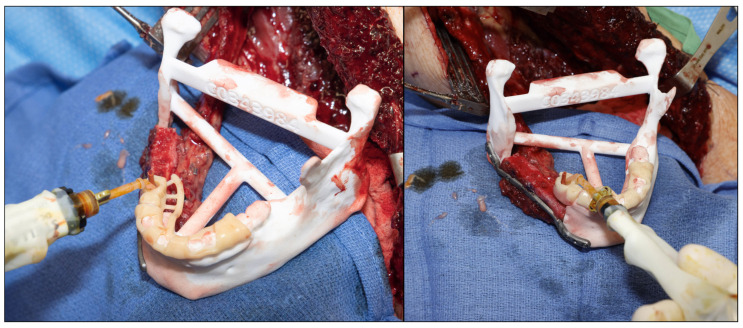
Temporary denture pick-up process. Note the opening of the temporary coping abutment, sealed with a red rubber catheter to prevent inadvertent acrylic flow into it.

After confirming the passive position of the temporary prosthesis and the fibula segments, the prosthesis pickup is performed. The authors use Stellar DC Acrylic, a dual-cure Poly Methyl Methacrylate (PMMA) pickup acrylic with a light-curing time of 20 s and a self-curing time of 120 s. The dual-curing feature enables the reconstructive team to use the acrylic without the need for a curing light in the operating room. The acrylic tube is enclosed in a sterile plastic bag, allowing intraoperative application while preserving sterility. The application tips are submerged in povidone-iodine for 10 min before use. Next, the acrylic is applied around the temporary coping abutments, taking care to avoid injecting the acrylic into the opening of the temporary coping abutments. It is then left to self-cure (Figure 13). After 120 s, the red rubber catheter is removed, the temporary coping retaining screws are removed, and the prosthesis is removed from the flap. At this point, the temporary prosthesis is bonded to the temporary coping abutments, thereby maintaining the spatial position of the implants in the fibula.

**Figure 14:**
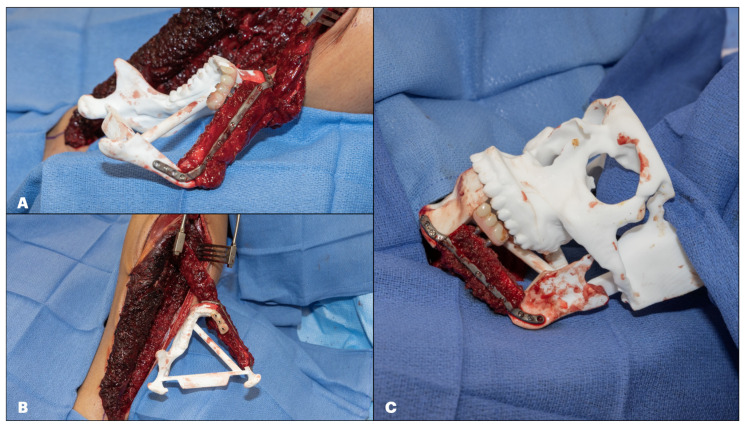
(**A**): Verification of the vertical position of the denture on reconstruction. (**B**): Verification of the transverse position of the denture. (**C**): Verification of occlusion with articulated maxillary model.

Once the stability and complete curing process of the acrylic have been verified, the arms of the floating prosthesis are removed using a pineapple rotating bur, and the sharp edges are trimmed down. Any remaining space between the denture and coping abutment is filled with acrylic and left to cure. Once this is complete, the temporary denture is placed again over the multi-unit abutments, now without the floating part, and the temporary coping abutments are secured to the fibula with the retaining screws. At this point, the position of the implants is verified on the resected model, and the articulated maxilla model is used to check the occlusion (Figure 14).

After verifying the position of the implants and the reconstruction, the resected model is removed. The construct, comprising the flap, implants, reconstruction plate, and prosthesis, is then ready to be transferred to the neck.

**Figure 15:**
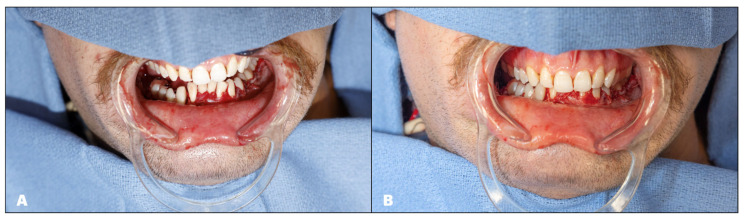
Jaw in a day after flap inset. (**A**): Temporary denture on the right posterior mandible. (**B**): Appreciate the small occlusal gap between the denture and the native maxillary dentition on the Right.

The flap is inset into the defect, the arterial and venous anastomosis is performed in the standard fashion, and the intraoral closure is finalized. Closing the mucosa or skin paddle under the temporary prosthesis is a challenging yet crucial step to prevent salivary leakage. The authors recommend using horizontal mattress sutures to encircle the multi-unit abutments and cinch the tissue to the implants. In some cases, a parachuting technique is employed, where all the sutures are initially placed and then tied at the end to allow for more space during the suturing process. At the end of the case, the occlusion is verified, and any premature contacts are removed to avoid occlusal contacts with the denture. (Figure 15).

### 3.3. Post-Operative Care

As with any other microvascular flap, post-operative care following JIAD reconstruction is critical to ensure flap viability, implant integration, and prosthetic hygiene. Flap monitoring adheres to standard microsurgical protocols, which involve daily assessments of perfusion, skin paddle integrity (if present), and intraoral closure [10]. Patients are maintained with a nasogastric feeding tube for one to two weeks, until intraoral soft tissue closure is confirmed. At this point, the patients transition to a full liquid diet, followed by a soft, non-chewing diet for at least 4–6 weeks, to minimize functional loading of the implants. Prosthetic hygiene is reinforced through collaboration with the patient’s prosthodontic team, which will oversee the transition to a definitive dental rehabilitation once bone healing and dental integration are complete, approximately 6 months after the initial surgery.

## 4. Conclusions

JIAD surgery marks a groundbreaking advancement in mandibular reconstruction, delivering immediate functional and esthetic rehabilitation. While the technique may seem complex, it is entirely achievable with thorough preparation and strict adherence to this proven protocol. This guide serves as a crucial resource for early adopters, clearly outlining each step of the process and emphasizing critical technical and logistical considerations. With this roadmap, surgeons can confidently and precisely embark on their first JIAD reconstruction.

## Figures and Tables

**Table 1:** Data required for jaw in a day.

CT-SCAN	CT maxillofacial or neck with or without contrast. CTA bilateral lower extremities Slice thickness < 1.25 mm
DENTAL SCAN	Recommended: intraoral scan Impressions and table scan of cast models accepted
PREOPERATIVE PICTURES	Standard pre-operative pictures (Front, Profile, Smile)

## Data Availability

The original contributions presented in this study are included in the article. Further inquiries can be directed to the corresponding author.
